# Characterization of the binding of MRTX1133 as an avenue for the discovery of potential KRAS^G12D^ inhibitors for cancer therapy

**DOI:** 10.1038/s41598-022-22668-1

**Published:** 2022-10-22

**Authors:** Abdul Rashid Issahaku, Namutula Mukelabai, Clement Agoni, Mithun Rudrapal, Sahar M. Aldosari, Sami G. Almalki, Johra Khan

**Affiliations:** 1 Bio-computation and Drug Design Laboratory, School of Health Sciences, University of KwaZulu-Natal, Westville Campus, Durban, 4001 South Africa; 2 Department of Physiotherapy, School of Health Sciences, University of KwaZulu-Natal, Westville Campus, Durban, 4001 South Africa; 3 Department of Pharmaceutical Chemistry, Rasiklal M. Dhariwal Institute of Pharmaceutical Education and Research, Pune, Maharashtra 411019 India; 4 Department of Medical Laboratory Sciences, College of Applied Medical Sciences, Majmaah University, Al-Majmaah, 11952 Saudi Arabia

**Keywords:** Computational biology and bioinformatics, Drug discovery, Chemistry

## Abstract

The Kirsten rat sarcoma (KRAS) oncoprotein has been on drug hunters list for decades now. Initially considered undruggable, recent advances have successfully broken the jinx through covalent inhibition that exploits the mutated cys12 in the switch II binding pocket (KRAS^G12C^). Though this approach has achieved some level of success, patients with mutations other than cys12 are still uncatered for. KRAS^G12D^ is the most frequent KRAS mutated oncoprotein. It is only until recently, MRTX1133 has been discovered as a potential inhibitor of KRAS^G12D^. This study seeks to unravel the structural binding mechanism of MRTX1133 as well as identify potential drug leads of KRAS^G12D^ based on structural binding characteristics of MRTX1133. It was revealed that MRTX1133 binding stabilizes the binding site by increasing the hydrophobicity which resultantly induced positive correlated movements of switches I and II which could disrupt their interaction with effector and regulatory proteins. Furthermore, MRTX1133 interacted with critical residues; Asp69 (− 4.54 kcal/mol), His95 (− 3.65 kcal/mol), Met72 (− 2.27 kcal/mol), Thr58 (− 2.23 kcal/mol), Gln99 (− 2.03 kcal/mol), Arg68 (− 1.67 kcal/mol), Tyr96 (− 1.59 kcal/mol), Tyr64 (− 1.34 kcal/mol), Gly60 (− 1.25 kcal/mol), Asp12 (− 1.04 kcal/mol), and Val9 (− 1.03 kcal/mol) that contributed significantly to the total free binding energy of − 73.23 kcal/mol. Pharmacophore-based virtual screening based on the structural binding mechanisms of MRTX1133 identified ZINC78453217, ZINC70875226 and ZINC64890902 as potential KRAS^G12D^ inhibitors. Further, structural optimisations and biochemical testing of these compounds would assist in the discovery of effective KRAS^G12D^ inhibitors.

## Introduction

Kirsten Rat Sarcoma (KRAS) viral oncogene is the most studied of the Ras family of proteins (HRAS, NRAS and KRAS) with the most mutations that result in a range of cancers including colorectal cancer (CRC), non-small cell lung cancer (NSCLC) and pancreatic adenocarcinoma (PDAC)^1^. In these cancers, KRAS is responsible for approximately 45% of cases in CRC, 35% in NSCLC and 90% in PDAC with poor prognosis and resistance to standard-of-care chemotherapy, denoting a critically unmet medical need that requires novel therapies to target KRAS^2^. However, unlike other oncogenic proteins such as Vascular Endothelial Growth Factor Receptor (VEGFR) and Epidermal Growth Factor Receptor (EGFR) that are easily druggable^3,4^, drugging KRAS was hampered by the lack of a well-defined binding site and its high affinity towards an abundance of GDP/GTPin the cytosol until recently^5,6^.

KRAS rotates between a GTP-bound and GDP-bound states representing an active and an inactive states respectively whose conversion is controlled by GEFs which produce the exchange from GDP to GTP, and GAPswhich influence the hydrolysis of GTP^7,8^. The activation by binding GTP perturbs the effector protein binding regions of KRAS by altering their conformations^9^ which resultantly activate downstream signaling pathways^10,11^. The mutations at codon 12 to other residue with a wide steric footprint hinders the binding of KRAS with GTPase activating proteins (GAPs) thereby interfering with GTP hydrolysis^12^. This interference locks KRAS in its innately active GTP-bound state hence favoring KRAS effector pathways activation^13^. Being a cellular signal transducer, the activation of KRAS biologically affects three downstream signaling pathways such as the rapidly accelerated fibrosarcoma-mitogen-activated protein kinase kinase-extracellular signal-regulated kinase (RAF-MEK-ERK)^14^, RAL guanine nucleotide dissociation stimulator-RAL (RALGDS-RAL)^15^ and phosphoinositide 3-kinase-protein kinase B-mammalian target of rapamycin (PI3K-AKT-Mtor)^16^. Responding to other stimuli and growth factors, KRAS together with its downstream pathways regulate cellular functions including survival, proliferation, migration, differentiation, adhesion and apoptosis which becomes corrupted upon mutations of KRAS resulting in over activation and cancer^17^. These mutations which mostly occurat codon 12, 13 and 61 ( G12D, G12V,G12C, G13D and Q61R) accounting for 70% of cases with missense mutation at codon 12 and 13 limiting the interaction of the protein with GAPs thus preventing the switching off of KRAS^18^. Due to the reasons stated in the first stanza, it took several decades of research to finally drug KRAS. However, this breakthrough is limited to G12C wherein the inhibitory activity depends on an electrophilic warhead forming a stable covalent bond with the nucleophilic head of the mutated cysteine^19^ thus these inhibitors occupy the induced switch II pocket^20,21^. This selective targeting of KRAS^G12C^ has left KRAS cancer patients driven by other mutations with unmet medical needs, spurring further search for inhibitors with novel mechanism of action to reversibly inhibit KRAS. KRAS^G12D^ is the most common occurring mutation among the KRAS driven tumors representing 33% of cases^18^ and has therefore been on the top list of drug hunters, however, due to the absence of a reactive residue within the binding pocket, developing inhibitors has been challenging wherein limited cellular activities were observed^22–24^. Nonetheless, the constant perseverance through optimization and structure-based activity improvements by drug developers has yielded MRTX1133, a reversible, potent and selective KRAS^G12D^ inhibitor which has shown efficacy in KRAS^G12D^ mutant xenograft mouse tumor model^25^.

**Figure 1:**
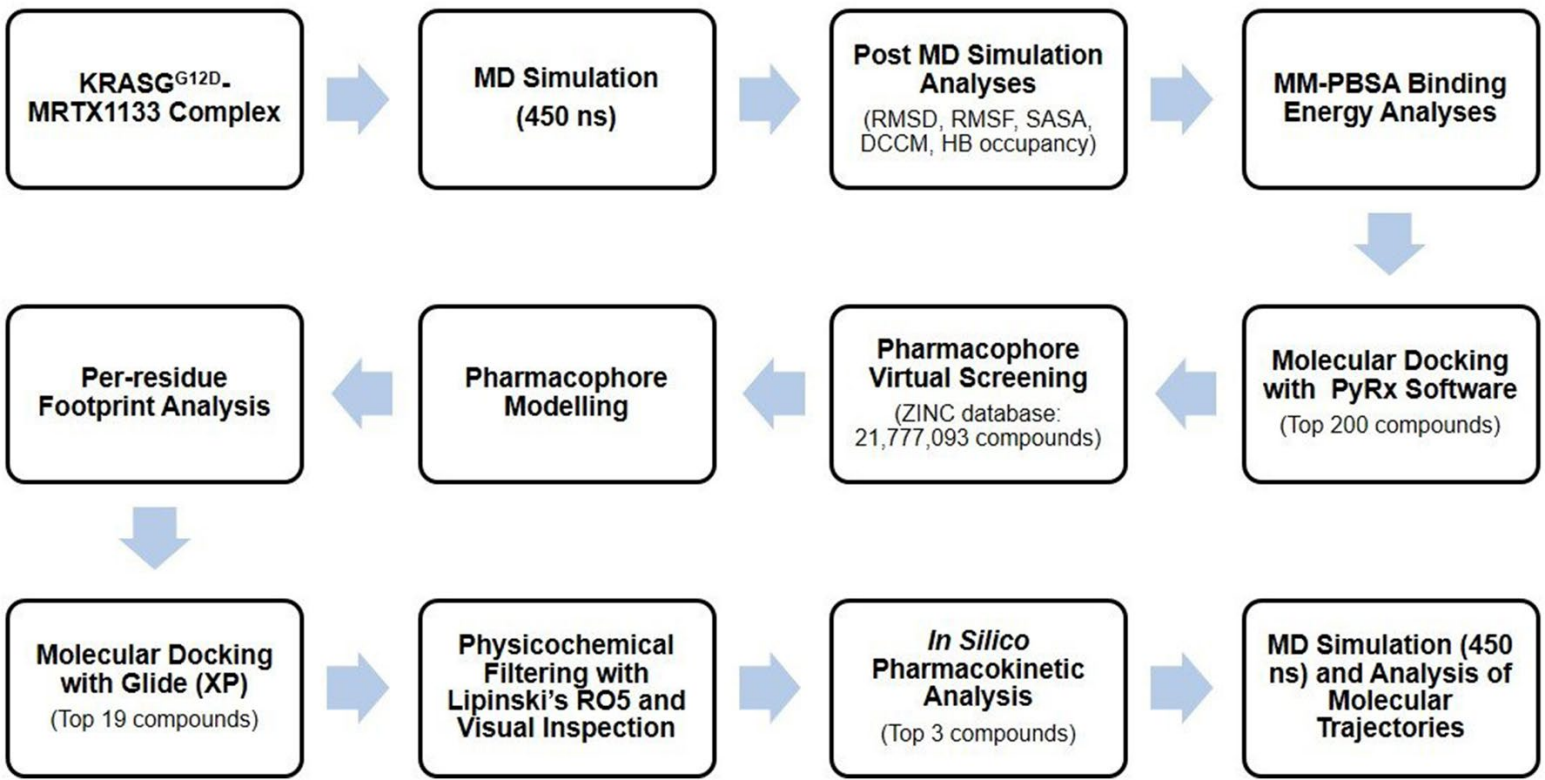
Schematic diagram of the approach applied to this study. First four sections indicate the characterization approach applied to MRTX1133, while rest of the sections depict the process of hit compounds identification.

In this current study, we sought to investigate and provide molecular insights and conformational dynamics bases for MRTX1133 evasion of KRAS^G12D^ resistance through molecular dynamics simulations and structural analysis (Fig. 1). These insights would be further exploited for the design of structure-based pharmacophore towards the identification of hit compounds with KRAS^G12D^ inhibitory potentials from the ZINC database.

## Methodology

### System preparation

The x-ray diffraction crystal structure with 1.3 Å resolution in complex with MRTX1133 and GDP was retrieved from the protein data bank (PDB ID: 7RPZ)^26^. The retrieved protein was prepared using UCSF Chimera candidate version 1.13.1^27^ by removing all nonstandard residues including water molecules. The prepared complex, which included the KRAS^G12D^, MRTX1133 and the GDP was saved as a KRAS^G12D^-MRTX1133 complex. The protein containing only the GDP was also saved as holo. The systems (KRAS^G12D^-MRTX1133 and holo protein) were then prepared for MD simulation by adding amber charges to MRTX1133 and the GDP, while all atoms of the protein complexes were minimized using the protein preparation wizard of Schrödinger Maestro (*Schrödinger Suite 2017–4 Protein Preparation Wizard*, 2018).

### Molecular dynamics (MD) simulations

The systems set up were taken through a long-range molecular dynamics simulation. The graphical processing unit version of Particle Mesh Ewald Molecular Dynamics (PMEMD) engine in AMBER 18 package^28^ was used to perform the simulation. The AMBER force field (FF14SB)^29^ and the integrated pdb4amber program were used to parameterize and modify the proteins, respectively, while the GDP and MRTX1133 were parameterized using the antechamber module of AMBER18. This module was also used to create partial atomic charges for the ligands. Afterwards, the tLeap module of AMBER 18 was utilized to neutralize the systems by adding Na^+^ and Cl^−^ counter ions as well as solvating the systems by suspending them in Transferable Intermolecular Potential with 3 Points (TIP3P) water box of 10 Å. Input coordinates and topology files for the systems were then generated using the tLeap module. The systems were then initially minimized for 2500 steps with the steepest descent and then switched to conjugate gradient algorithm for further 5000 steps minimization using 500 kcal/molÅ^−2^ restraint potential. Afterwards the systems were then heated gradually from 0 to 300 K for 5 ps in isothermal-isobaric NTP ensemble with Langevin thermostat at a pressure of 1 bar using Brendsen barostat^30^. Equilibrations were then performed to relax the systems at 300 K for 1000 ps without energy restraint. Molecular dynamics production was then performed for 450 ns with the SHAKE algorithm^31^ used to restrain all hydrogen bonds at 2 fs time step.

### Post MD analysis

The trajectories and coordinates generated from the MD simulations were all analyzed using the CPPTRAJ and PTRAJ modules^32^ of AMBER 18. Origin data tool^33^ was used to plot all graphs whiles visualization of snapshots was performed with Discovery Studio (BIOVIA 2017) and UCSF Chimera^27,34^.

### Thermodynamics estimations

We utilized the molecular mechanics Poisson-Boltzmann surface area (MM-PBSA) method to predict the binding free energy of the complexes using the mmpbsa.py module^35^ incorporated in AMBER 18. This method is widely used due to its efficiency and low cost. From each of the trajectories generated from the systems, the binding free energy was averaged over 30,000 snapshots using a time step of 50 ps. The energies calculated from this method are depicted as follows:

1 $$\Delta {\text{G}}_{{{\text{bind}}}} = {\text{ G}}_{{{\text{complex}}}} {-}{\text{ G}}_{{{\text{receptor}}}} {-}{\text{ G}}_{{{\text{ligand}}}}$$

2 $$\Delta {\text{G}}_{{{\text{bind}}}} = {\text{ E}}_{{{\text{gas}}}} + {\text{ G}}_{{{\text{sol}}}} {-}{\text{ T}}\Delta {\text{S}}$$

where ΔG_bind_ is taken to be the sum of the gas phase and solvation energy terms less the entropy (TΔS) term

3 $${\text{E}}_{{{\text{gas}}}} = {\text{E}}_{{{\text{int}}}} + {\text{E}}_{{{\text{vdw}}}} + {\text{E}}_{{{\text{ele}}}}$$

where E_gas_ is the total of the AMBER force field internal energy terms. E_int_ (bond, angle and torsion), the covalent van der Waals (E_vdw_) and the non-bonded electrostatic energy component (E_ele_). The solvation energy is denoted by the equation:

4 $${\text{G}}_{{{\text{sol}}}} = {\text{G}}_{{{\text{PB}}}} + {\text{ G}}_{{{\text{non}} - {\text{polar}}}}$$

5 $${\text{G}}_{{{\text{non}} - {\text{polar}}}} = {\text{ SASA}}$$

The polar solvation contribution is denoted as G_PB_ and G_non-polar_ represents the non-polar contribution energy and is computed from the solvent assessable surface area (SASA) which is obtained by the use of 1.4 Å water probe radius. Per-residue decomposition analyses were also performed to estimate individual energy contribution of the residues of the substrate pocket to the affinity and stabilisation of the compounds.

### Dynamic cross correlation matrices (DCCM)

Analysis of the dynamic cross correlation of MD simulation trajectory is used to investigate the correlated motion of residual-based fluctuations during the course of simulation. This investigation was performed on the two switches of the protein which are very critical for interaction with effector proteins. The cross-correlation coefficient C_ij_ for the pair of C–α atoms i and j were calculated based on the formula below:

6 $$C_{ij} = \frac{{\left\langle {{\Delta r}_{{\text{i}}}^{*} {\Delta r}_{{\text{j}}} } \right\rangle }}{{\left( {\left\langle {{\text{r}}_{{\text{i}}}^{2} } \right\rangle \left\langle {{\Delta r}_{{\text{j}}}^{2} } \right.} \right)^{{{\raise0.7ex\hbox{$1$} \!\mathord{\left/ {\vphantom {1 2}}\right.\kern-\nulldelimiterspace} \!\lower0.7ex\hbox{$2$}}}} }}$$

where Δr_j_ and Δr_i_ are the displacement from the mean locations of the *j*th and *i*th atom respectively. The cross-correlation coefficient C_ij_ has a range of + 1 to − 1 wherein the lower and upper limits denote fully anti-correlated and correlated movements during the course of simulation. The CCPTRAJ module incorporated in AMBER 18 package was used to calculate the dynamic cross-correlation.

### Principal component analysis (PCA)

We used the PCA approach to evaluate changes in the compound’s dynamics and conformations during the period of simulation in reference to the MRTX1133. This method comprises the eigenvalues and eigenvectors which respectively informs on the quantum and direction of motions. The two principal components were calculated by employing the CPPTRAJ module, whereas the conformational behaviours were projected along the directions of these two components (ev1/PC1 vs. ev2/PC2).

### Pharmacophore modelling

The phase model was utilized to generate a pharmacophore model centred on PRED analysis of the KRAS^G12D^-MRTX1133 complex. The residues that contributed energies above − 1.0 kcal/mol to the total free binding energy of MRTX1133 complexing were identified and their respective interactions with the moieties on MRTX1133 were used as the bases for the pharmacophore model.

### Virtual screening and docking of hits

The pharmacophore model created was then used for virtual screening of the ZINC database^36^ for inhibitors that possessed good fitness with RMSD values less than 0.9 Å. The retrieved inhibitors were then prepared for structure-based screening with PyRx software^37^ with a grid box of center: x = 0.48195, y = 3.92564, and z = − 22.5689 with size of: x = 20.183, y = 17.1422, and z = 18.9123. Compounds that showed binding energies lower than − 8.5 kcal/mol were eliminated whiles those scoring − 8.6 kcal/mol and above were further processed for docking with Glide^38^.The KRAS^G12D^ protein was prepared using the protein preparation wizard suit incorporated in Schrödinger. For the binding site, a grid box centred at MRTX1133 was utilized to cater for maximum length of the ligand. OPLS3e force field^39^ was applied to input partial charges and default van der Waals scaling was employed. The selected compounds were flexibly docked with extra precision (XP), with a maximum of writing out one pose per compound as well as post docking minimisation of the compounds. MRTX1133 was redocked and the docking conformation was realigned with its co-crystalized conformation to determine the root mean square deviation (RMSD) to validate the docking program.

### ADME and toxicity prediction

The pharmacokinetics of the hit compounds were analysed with SwissADME^40^, an online server that evaluates the hit compounds functionality inside the body. Evaluating the toxicity of compounds is also an important part of the drug development process. This allows for the determining of the toxic features of a compound through the estimated lethal dose (LD_50_) in mg/kg weight. ProTox-II, an online server to determine the toxicity of compounds by incorporating fragment propensity, machine learning and molecular similarity was utilized^41^. The compounds are classified into categories ranging from class I to VI based on their safety according to the globally harmonized system of classification of labelling chemicals^42,43^.

### MD simulation of hit compounds

The simulation of selected hit compounds was performed using the same protocol used for the KRAS^G12D^-MRTX1133 complex as explained above.

## Results and discussion

The KRAS protein and its other family members (HRAS and NRAS) consist of two main components, a membrane-targeting hyper variable region (HVR) and the conserved catalytic domain^44^. The conserved domain is made up of five alpha helices that surrounds six beta sheets and engages in interactions with regulators and effectors which is conditioned by conformational changes of two flexible regions. These flexible regions, switch I and Switch II cover residue 30–38 and 59–76 respectively^45^. We therefore investigated the impact of MRTX1133 on these switches.

### MRTX1133 binding stabilizes KRAS^G12D^ binding site

**Figure 2:**
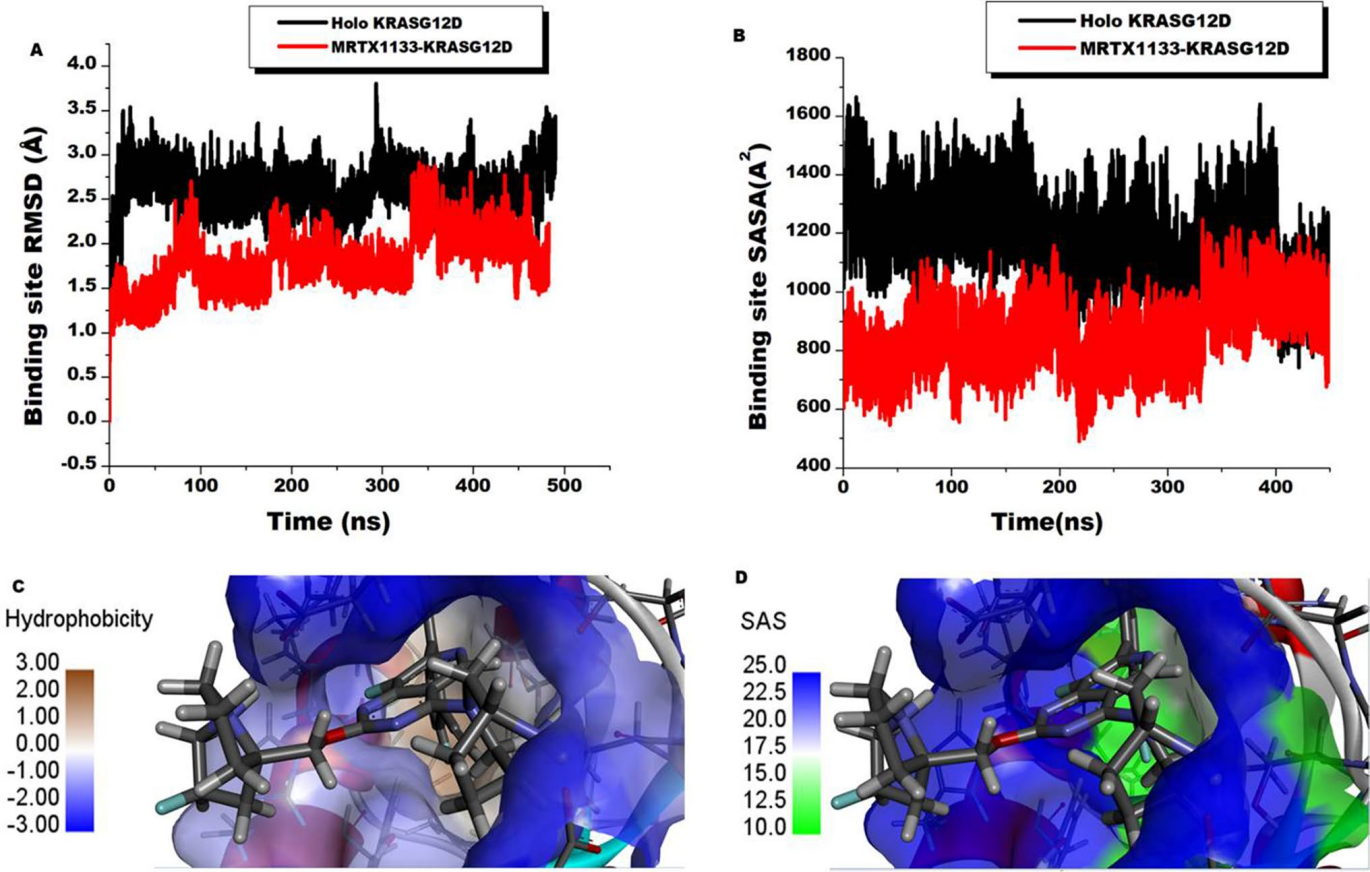
Comparative stability and solvent accessibility surface area of the binding site of KRAS^G12D^ upon the binding of MRTX1133 and the holo protein. (**A**) Shows the RMSD plots of the C-a atoms of the binding site residues indicating MRTX1133 binding stabilized the residues (red) relative to the holo (black). (**B**) Shows the SASA plots of the binding site residues indicating a reduction in surface area availability upon MRTX1133 binding (red) relative to the holo protein (black). (**C**) Shows the hydrophobicity of the binding site. (**D**) Shows the solvent accessible of area of the binding site.

Binding allosterically to KRAS^G12D^, MRTX1133 effectiveness as a small molecule inhibitor is to instigate a communication across the structure to a spatially distant site specifically the switches (I and II) whose conformational changes influence the catalytic domain’s interactions with effector and regulatory proteins^46^. This communication will depend on the immediate impact of the small molecule on the binding pocket of the protein. Thus, we first sought to determine the effect of MRTX1133 binding on the allosteric binding pocket residues by computing the deviations of the C–α atoms of the pocket residues over 450 ns simulation relative to the starting structure. The root-mean square deviation (RMSD) matric was therefore utilized and the results displayed in Fig. 2. The occupied site showed an average RMSD values of 1.80 Å, while the unoccupied pocket displayed RMSD values of 2.61 Å. These results indicate MRTX1133 presence induced stability of the pocket residues, a deviation from the normal state. An analysis of the solvent accessibility surface area (SASA) of the pocket was also investigated and shown in Fig. 2. Average SASA results of 1169.66 A^2^ and 856.44 A^2^ were presented by the holo and MRTX1133 sites respectively. This matric is informative of the surface area of the pocket that is exposed to solvent interaction and thus could also be indicative of the folding and/or realignment of the residues towards the hydrophilic or hydrophobic core of the protein. As observed, the presence of MRTX1133 reduced the surface area of the site that is available for external interaction in a holo state by 313.22 A^2^. Thus, the site residues favored the hydrophobic core of the protein in MRTX1133 presence. The hydrophobicity as presented in Fig. 2C estimates the degree of affinity between the residues side chains and water. This feature is critical for the stabilization and folding of the protein structure^47^. As observed, the deeper the residues in the pocket the higher the hydrophobicity. This is depicted by the color bar wherein high hydrophilicity is denoted by blue color whiles hydrophobicity is brown. Taken together, the stability and the increase in hydrophobicity of the site residues could condition signaling to the protein–protein interface that influences its overall therapeutic effect.

Since proteins are sensitive to stimuli, these induced changes could be the initiation process for the inhibition of KRAS. To further ascertain the impact of these changes on distant critical domains, we investigated the residual response to these pocket site changes.

### KRAS switches reacts to binding site changes through correlated residual movements

**Figure 3:**
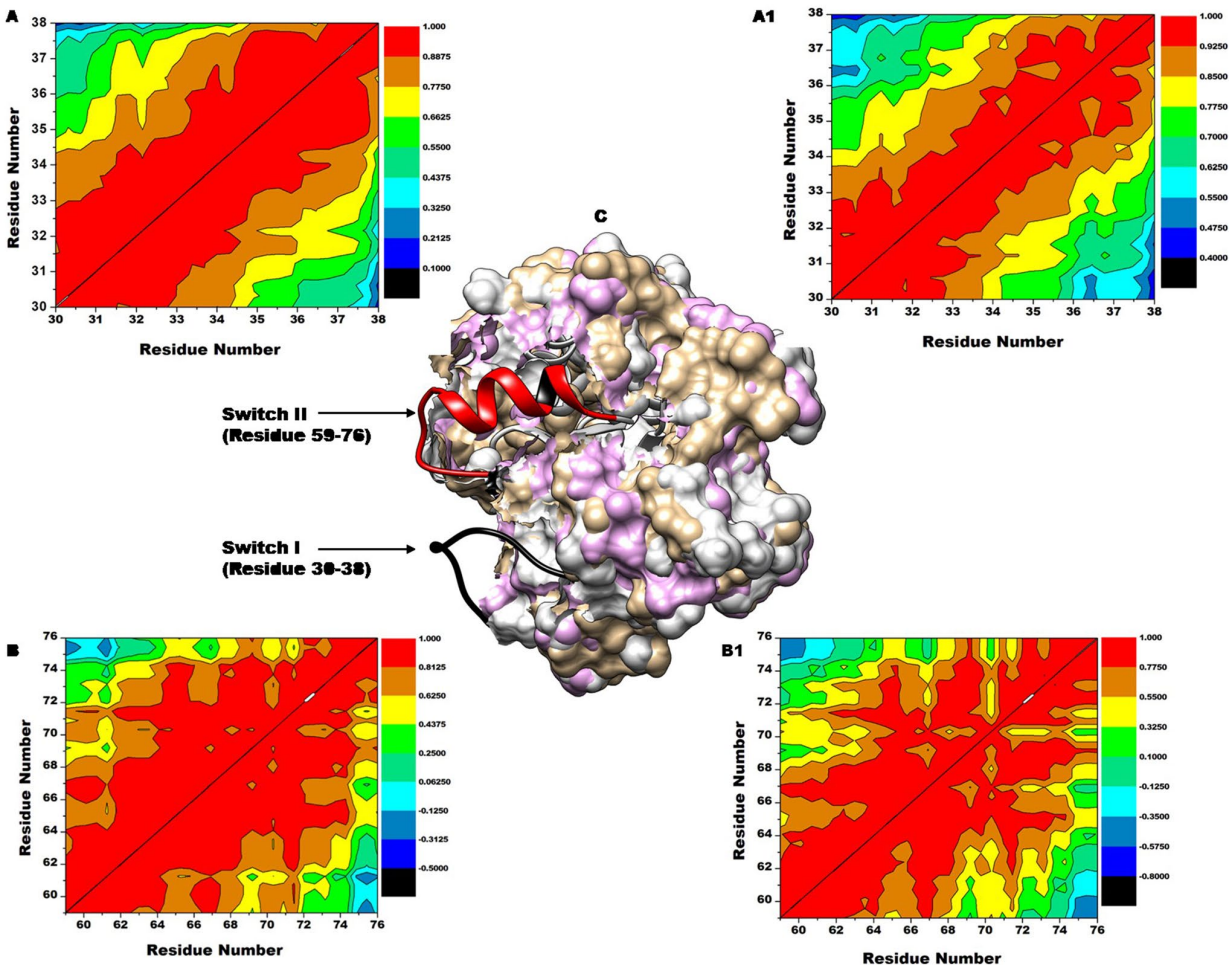
Cross-correlation matrices of the fluctuations of coordinates of C–a atoms of the switches around their mean positions during the 450 ns simulation. DCCM plots of switch I MRTX1133 complex (**A**), Switch I holo (**A1**), Switch II MRTX1133 complex (**B**), and (**B1**) Switch II holo protein. This indicates MRTX1133 complexing induced a more corelated movement. (**C**) KRAS structure showing switch I (black) and switch II (red).

The variations in internal correlation movements of the residues of switch I and II were analyzed by cross-correlation matrices of the C–α atoms fluctuations. Figure 3 shows the DCCM plots of switch I and II. The most negative regions (blue–black) and the most positive regions (yellow–red) reflect strong anti-correlated movements and strong correlated motions of the specific residues respectively. Juxtaposing the movements of switch I in a complexed state (Fig. 3A) and the holo (Fig. 3A1) shows a more correlated movement of the residues of the complexed switch than the holo as evidenced by the degree of red regions presented. This trend was observed in the comparison of switch II of the complexed state (Fig. 3B) and the holo switch II (Fig. 33B1). Generally, the binding of MRTX1133 presented strong correlated residual movements of Switch I and II than the unbound (holo) over the period of simulation. This is observed from the degree of positive (red) regions depicted by the Fig. 3. This differential observation could influence the switches interaction with effector and regulator proteins thus highlighting the basis of MRTX1133 inhibitory prowess.

### Atomistic interactions underscoring the potency of MRTX1133

**Figure 4:**
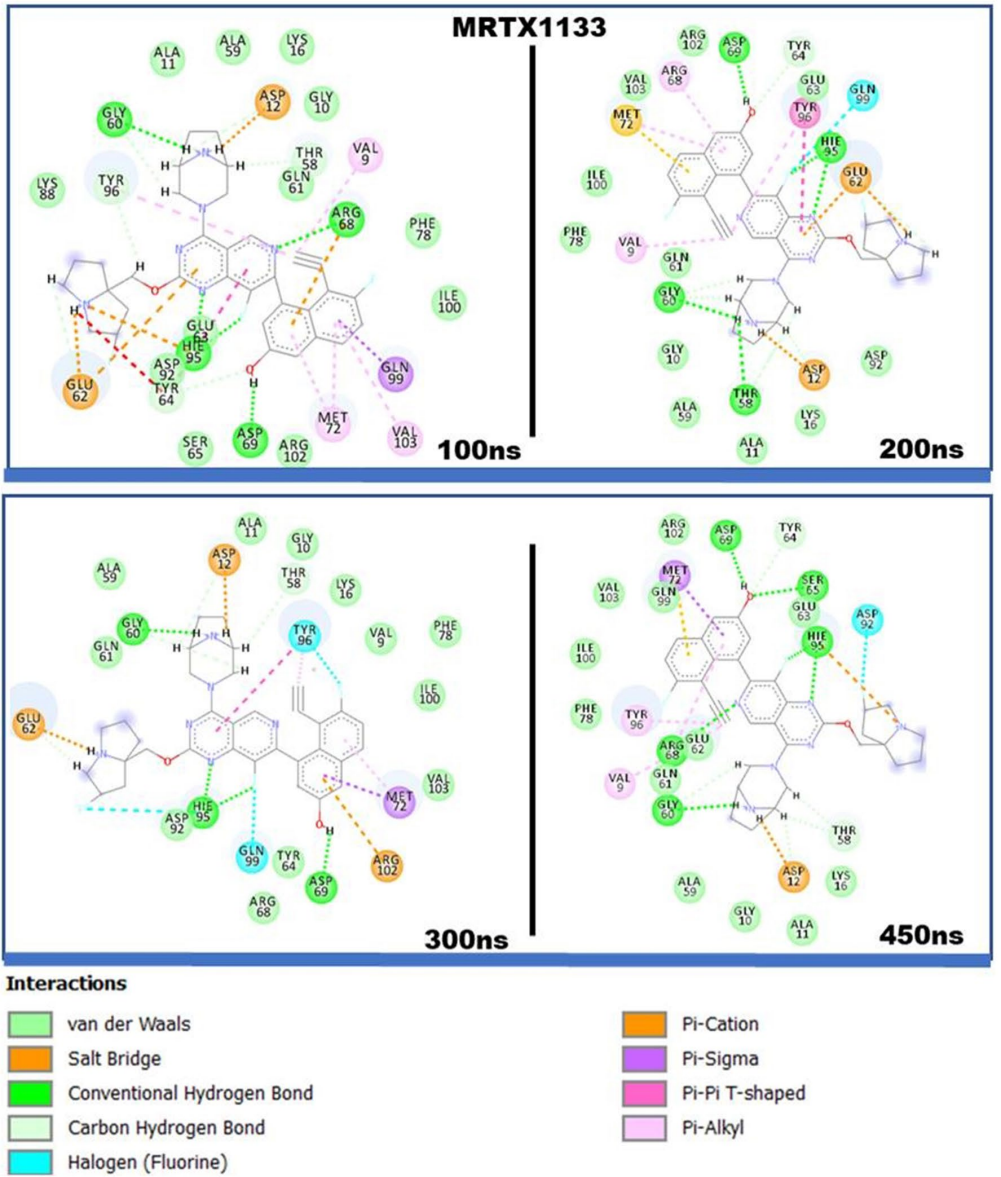
2D interactions of MRTX1133 with KRAS^G12D^ binding site residues. Snapshots depicts interactions at 100 ns, 200 ns, 300 ns and 450 ns. MRTX1133 engages in varying interactions.

Upon attaining the impact of the binding of MRTX1133 on the structural dynamics of the protein, we further probed at the atomic level to ascertain the underlying interactions that accounts for the observable structural changes. Thus, six snapshots were taken from the 450 ns simulation trajectory for analysis. Trajectories generated during the process of simulation contain events that helps to unravel structural activities and atomistic insights that occurred at a specific time-frame^48^. Snapshot visualization analysis revealed MRTX1133 formed varied bond types with the binding site residues. While some of these interactions endured though the 450 ns simulations, others were altered during the process as observed in Fig. 4. The sp^2^ hybridized O atom of Gly60 was observed to form a conventional hydrogen bond with the HNO3 atom of MRTX1133in all the observed snapshots. Asp69 and His 95 were also observed to form an enduring hydrogen bond with the compound. The sp^2^ hybridized OD1 atom of Asp69 interacted with the H02 atom of MRTX1133, while the HE2 atom of His95 interacted with both the sp^2^ hybridized NO1 and sp^3^ hybridized F01 atoms of MRTX1133. Thr58 interacted with the HN03 atom of the compound through the sp^2^ hybridized O atom, which was observed at 100 ns, 200 ns, and 300 ns. Arg68 was also observed to interact through conventional hydrogen bond through the HH21 atom and the sp^2^ hybridized N03 atom of MRTX1133 at 200 ns and 300 ns, while Ser65 was observed to interact at only 300 ns through the H atom and the sp^3^ hybridized O02 atom. To highlight the percentage of time occupied by these interactions in the 450 ns simulation period, the hydrogen bond occupancy was calculated and presented in Table 1. The hydrogen bond occupancy captures hydrogen interactions including those occurred outside the trajectory captured by the snapshots.

**Table 1:** Dynamic hydrogen bond interactions of MRTX1133 at KRAS^G12D^ binding site over the 450 ns MD simulation.

Compounds	H-acceptor	H-donor	Occupancy (%)	Average distance (Å)
MRTX1133	ASP69-OD1	MRTX1133-HO02	99.7	2.59
	ASP12-OD1	MRTX1133-HN02	65.4	2.80
	GLY60-O	MRTX1133-HN03	53.9	2.82
	GLU62-OE1	MRTX1133-H06	50.1	2.79
	THR58-O	MRTX1133-HN03	43.4	2.80
	MRTX1133-N01	HIS95-HE2	39.9	2.91
	MRTX1133-N03	ARG68-HH11	20.5	2.91
	MRTX1133-F01	GLN99-HE22	10.2	2.88
	GLU63-O	MRTX1133-H02	5.0	2.90
	MRTX1133-O02	SER65-H	2.5	2.74
	MRTX1133-N01	TYR64-HH	0.9	2.95
	ALA59-O	MRTX1133-HN03	0.7	2.87
	MRTX1133-N02	TYR96-HH	0.5	2.83
	MRTX1133-F02	LYS88-HZ3	0.2	2.90

### Thermodynamics of MRTX1133

The binding of a ligand to proteins underpins a variety of recognition processes in biological systems. Among these range of processes is the binding energy which underscores the affinity of the ligand and receptor. The free binding energy therefore provides information on the spontaneity, ligand permeation and reaction kinetics^49^. The free binding energy profile of MRTX1133 was therefore estimated using the MM-PBSA method as outlined earlier and presented in Table 2. MRTX1133 showed total free binding energy (ΔG) of − 73.16 kcal/mol. This low binding energy is indicative of its spontaneity and permeation in to the hydrophobic core of the protein thus engaging in many interactions with the site residues which result in more release of energy. This observation could therefore explain the low pERK IC_50_ of 2 nM recorded experimentally for MRTX1133 thus its observed potency. The gas phase, electrostatic and van der Waals interaction were observed as prominent contributors to the total free binding energy.

**Table 2:** Binding energy profile of MRTX1133 compounds.

System	Energy components (kcal/mol)				
	${\Delta E}_{{{\text{vdw}}}}$	${\Delta E}_{{{\text{ele}}}}$	${\Delta G}_{{{\text{gas}}}}$	${\Delta G}_{{{\text{sol}}}}$	${\Delta G}_{{{\text{bind}}}}$
KRAS^G12D^-MRTX1133	− 62.40 ± 4.73	− 633.03 ± 30.37	− 695.43 ± 29.75	622.27 ± 29.75	− 73.16 ± 5.15

ΔE_ele_ = electrostatic energy; ΔE_vdW_ = van der Waals energy; ΔG_bind_ = total binding free energy; ΔG_sol_ = solvation free energy ΔG = gas phase free energy.

**Figure 5:**
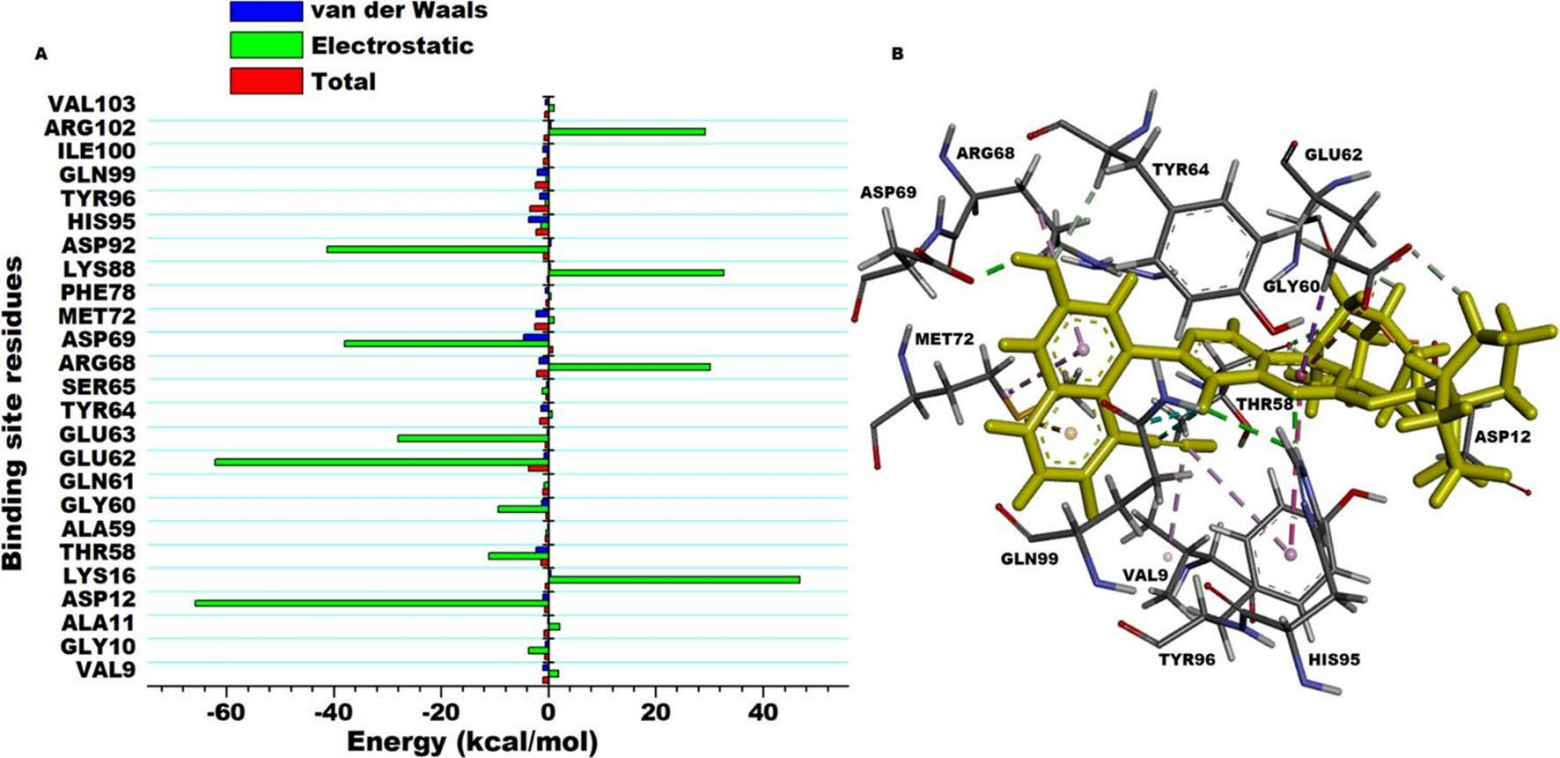
Per-residue decomposition analysis showing binding site residue energy contributions towards the complexing of MRTX1133. Electrostatic energy featured prominently towards the total free binding energy. (**A**) Shows the energy plots. (**B**) Shows the interacting residues.

Since the binding interactions between the ligand and the binding site residues is crucial to the quantum of energy released (total free binding energy), we decomposed the total free binding energy into individual residue contributions. This allowed us to identify the residues that individually contributed the most towards the complexing and stability of MRTX1133. The results are presented in Fig. 5. Asp69 (− 4.54 kcal/mol), His95 (− 3.65 kcal/mol), Met72 (− 2.27 kcal/mol), Thr58 (− 2.23 kcal/mol), Gln99 (− 2.03 kcal/mol), Arg68 (− 1.67 kcal/mol), Tyr96 (− 1.59 kcal/mol), Tyr64 (− 1.34 kcal/mol), Gly60 (− 1.25 kcal/mol), Asp12 (− 1.04 kcal/mol), and Val9 (− 1.03 kcal/mol) were observed as residue with prominent contributions. Others include: Ile100 (− 0.98 kcal/mol), Glu62 (− 0.73 kcal/mol), Arg102 (− 0.53 kcal/mol), Gly10 (− 0.50 kcal/mol), Val103 (− 0.48 kcal/mol) and Phe78 (− 0.48 kcal/mol) as depicted in Fig. 4. We therefore monitored these residues interactions with MRTX1133 over 450 ns simulation period to ascertain the functional groups of the ligand they engaged with.

### Pharmacophore model generation

**Figure 6:**
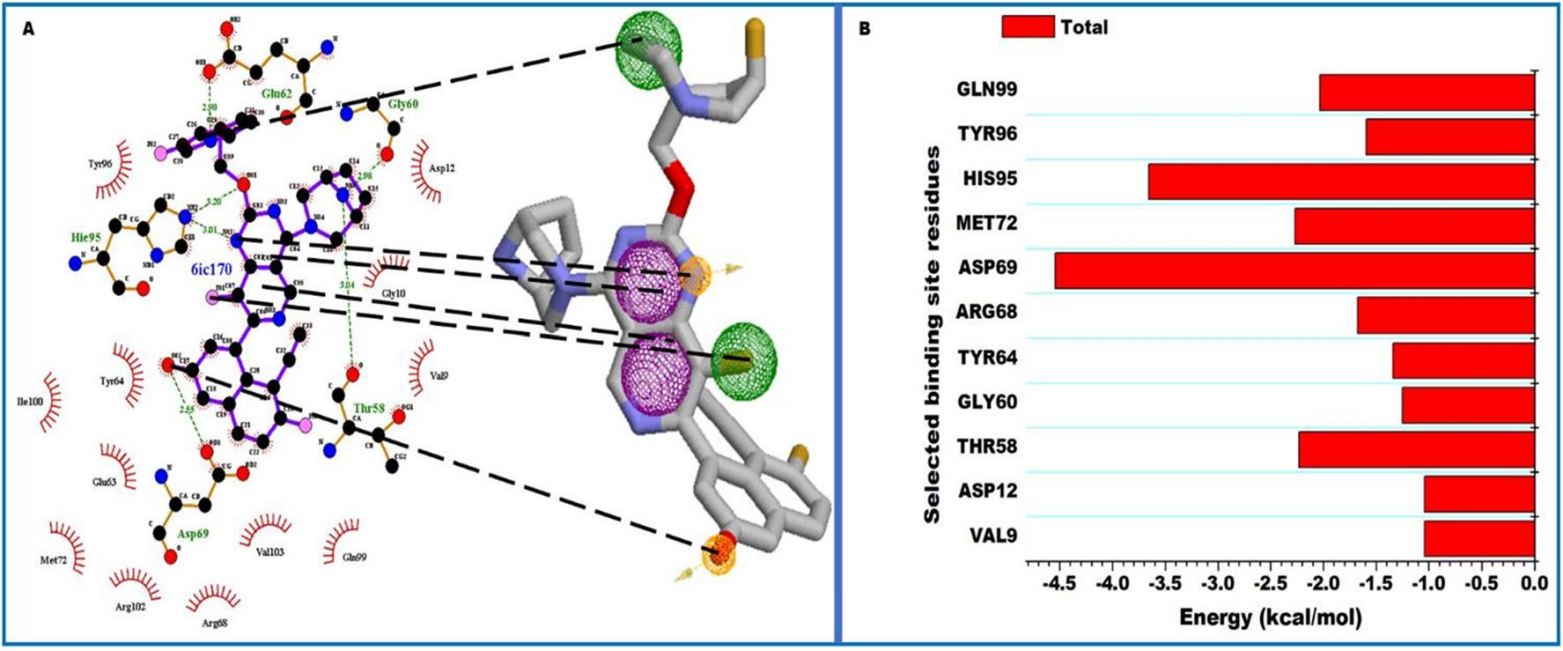
A pharmacophore model of MRTX1133. (**A**) 2D illustration of MRTX1133 interacting with binding site residues after 450 ns MD simulation. (**B**) The selected pharmacophoric moieties in MRTX1133, which interacted with the most negative energy contributing binding site residues. (**C**) PRED plot highlighting only the total binding energy contributed by the selected active site residues.

The functional or structural components of a bioactive compound that possess biological activity is often referred to as pharmacophores. These elements define important, steric and electronic function-determining points necessary for an optimal interaction with a pharmacological target, thus they represent a common denominator of a class of compounds that exhibit a similar pharmacological profile and are therefore recognized by the same site of the target protein^50^. A pharmacophore should therefore satisfy the criterial of interacting with crucial binding site residues. As such, we therefore identified the components of MRTX1133 that interacted with the residues that resulted in high energy contributions as crucial elements for pharmacophore modelling. Per-residue decomposition (PRED)-centered pharmacophore model was therefore generated from the 450 ns simulations performed for MRTX1133. Residues with contributions higher than − 1 kcal/mol to the total free binding energy of MRTX1133as presented above were monitored for the period of simulation to identify functional groups they engaged with. These crucial functional groups on MRTX1133 and their interactions is shown in Fig. 6. The functional groups were then used to create a pharmacophore model for compounds screening in ZINC database.

### Pharmacophore based virtual screening

The pharmacophore hypothesis generated was used as a 3D structural query with several combinations. The ZINC database was therefore screened for matching compounds. On zincpharmer, the 2 aromatic rings of the pyridopyrimidine, 2 hydrogen acceptors and 4 hydrophobic moieties were selected due to their interaction with the high energy contributing residues. Molecular weight ≤ 500 Da, rotatable bonds ≤ 10 were set as pre-requisite for the generation of ideal hits. A total of 21,777,093 compounds were screened. An initial 200 compounds were retrieved. These hit compounds were then docked into the allosteric binding site occupied by MRTX1133 using PyRx software and the compounds exhibiting high docking scores were further selected. Nineteen (19) compounds were selected and further subjected to docking using GLIDE docking suit incorporated in Schrödinger with an extra precision (XP). Three compounds were then selected from the compounds for further analysis. The results of the compounds are presented in Table 3 and in Table S1.

**Table 3:** Summary of binding energy scores obtained from PyRx and Glide XP for the identified top four compounds.

ZINC Compound ID	Pyrx docking score (kcal/mol)	Glide XP Score (kcal/mol)	2D Structure
ZINC78453217	− 10.1	− 9.77	
ZINC70875226	− 10.6	− 9.34	
ZINC64890902	− 9.8	− 10.0	
MRTX1133	− 11.4	− 10.92	

### Hit compound interactions within the binding pocket

**Figure 7:**
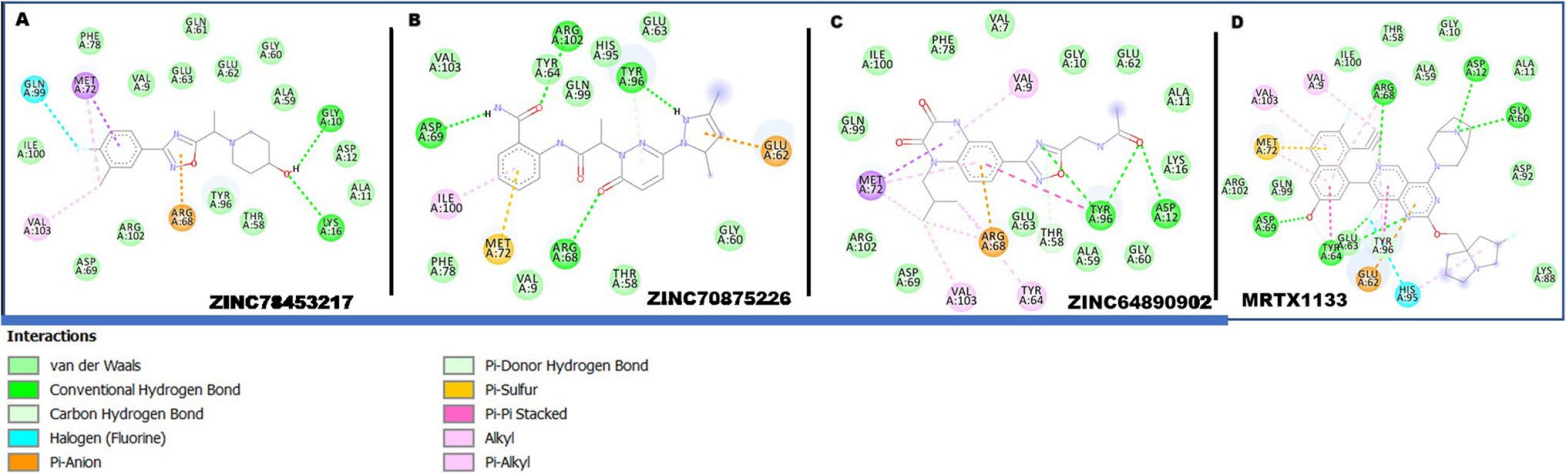
2D molecular interactions of MRTX1133 and selected compounds within the binding site of KRAS allosteric binding site. The selected compounds and MRTX1133 show similar interactions with the binding site residues suggesting the selected compounds have the potential to elicit similar therapeutic effects.

After the docking of the compounds, the interactions that characterized their binding was further investigated. The type of interactions exhibited by a compound in the binding site of a protein is crucial in determining it therapeutic influence on the protein^51^. After docking the selected compounds, we sought to ascertain the interactions that feature between the compounds and the binding site residues. We thus visualized the complexes in comparison to MRTX1133. The known inhibitor, MRTX1133, was observed to engage in differential interactions with the site residues. Key among the interaction types are conventional and carbon hydrogen interactions, pi anon and alkyl interactions, van der Waals and halogen interactions. These interactions between the inhibitor and the residues varied depending on the functional groups. The selected compounds were also observed to interact with the site residues through similar interaction types as presented in Fig. 7.

### Physicochemical properties and drug-likeness of the selected hits

To ascertain information into the drug-likeness of the hit compounds and their pharmacokinetic properties, a comparative analysis between the compounds and MRTX1133 was performed. The physicochemical properties of the three hit compounds and the other selected compounds were evaluated and presented in Tables 4 and S2 respectively. SwissADME, an online server was employed to profile their physicochemical properties.

**Table 4:** Physiochemical properties of the top 3 hit compounds relative to MRTX1133.

Physiochemical properties	Compounds			
	ZINC78453217	ZINC70875226	ZINC64890902	MRTX1133
Formula	C16H20FN3O2	C19H20N6O3	C17H19N5O4	C33H33F3N6O2
MW (g/mol)	305.35	380.40	357.36	602.65
MLog *P*_o/w_	2.66	1.46	1.48	3.25
LogS (Ali) (mol/L)	− 3.42	− 3.13	− 2.71	− 5.44
Class	Soluble	Soluble	Soluble	Moderately soluble
TPSA (A^2^)	62.39	124.90	122.88	109.42
Molar Refractivity	84.32	103.55	95.39	173.75
H-bond acceptors	6	5	6	8
H-bond donors	1	2	2	3
Rotatable bonds	3	6	6	5
Lipinski violations	No; 0	No; 0	No; 0	Yes; 1
**Toxicity**				
LD_50_ (mg/kg)	^1000^	^200^	^649^	^2500^
Toxicity class	^4^	^3^	^4^	^5^

^a^HBD = number of hydrogen bond donors (HBD ≤ 5); HBA = number of hydrogen bond donors (HBD ≤ 10); TPSA = total polar surface area; MLOGP = predicted octanol/water partition coefficient (MLOGP < 4.15); MW = molecular weight (g/mol) (MW ≤ 500); Rotatable bonds ≤ 8; TPSA = Topological polar surface area (≤ 140).

Generally, an increase in the molecular weight of a therapeutic agent reduces its concentration at the intestinal epithelial surface and hence decreases absorption^52^. Studies have, therefore, reported that this could prevent passive diffusion of the agent across the bilayer membrane. Thus, it is suggested that the molecular weight of drug candidates should be less than 500 g/mol in accordance with the Lipinski’s rule of five (RO5)^53,54^. The selected compounds together with the hits all possessed molecular weight within the acceptable range for drug-like molecules as presented in Tables 4 and S2. Interestingly, these compounds outscored MRTX1133 regarding the molecular weight suggesting they would possess better intestinal absorption and cellular uptake.

Also, to determine the impact of molecular weight on bioactivity is the number of rotatable bonds, an excellent definition of molecular flexibility^55^ (Lu et al. 2004). Verber et al.^56^ reported that the number of rotatable bonds increases with an increase in molecular weight. According to the Lipinski’s RO5, the number of rotatable bonds of a drug-like compound should be less than 10; this enhances absorption and bioavailability^55,57,58^. As observed from our evaluation, all the compound possessed rotatable bonds below 10 including MRTX1133 which possessed 5 rotatable bonds. LogP is the logarithm of the coefficient of chemical compounds’ permeation across n-octanol and water (C_octanol_/C_water_) and thus estimates the hydrophilicity of chemical compounds^59^. High MLogP figure depicts a reduction in aqueous solubility and this could result in reduction of compound absorption. The hit compounds all showed lower MLogP values lower than MRTX1133 (Table 4). Again, LogS which is indicative of membrane permeability was used to evaluate the aqueous solubility of the selected and hit compounds in comparison to MRTX1133. An acceptable range of 0 to − 6 for aqueous solubility accounts for 95% of approved drugs^60^. The compounds all fell within the acceptable range and are therefore classified as soluble while MRTX1133 was classified as moderately soluble.

The topological polar surface area (TPSA) strings the oxygen and nitrogen (surface polar atoms) in relation with the hydrogen atoms that are attached in a compound. It helps in determining the potential of a compound to traverse the cells wherein a low TPSA value depicts the compound can permeate the cell^61,62^. It also offers information on the volume and molecular size which controls a compound’s physiological transport across the lipid bilayer including the blood–brain barrier (BBB) and gastrointestinal tract (GIT)^63^. Elevated TPSA has been reported to interfere with the transportability of potential drugs which therefore reduces their bioactivity^64^. As presented in Table 3, two compounds, ZINC70875226 and ZINC64890902 exhibited TPSA values higher than MRTX1133 whiles ZINC78453217exhibited the least. All compounds however fell well with the acceptable range of TPSA of ≤ 140 Å^2^.

Hydrogen bond (HB) is one of the key factors that have been suggested to influence the solubility of therapeutic agents since they have to be severed to accelerate the transportation of the agents across the lipid bilayer membrane^65^. Therefore, a high number of HB affects permeation by passive diffusion as a result of the reduction in partitioning from the aqueous environment into the lipid bilayer membrane. Hydrogen bond acceptors (HBA) and hydrogen bond donors (HBD) are therefore indicators of hydrogen bonding of a compound. According to the Lipinski’s RO5, drug-like compounds should possess HBA ≤ 10 and HBD of ≤ 5 as the basis for oral bioavailability and activity^52^. The results computed in Tables 4 and S2 shows that all selected and hit compounds qualify for adequate hydrogen bond acceptance and donation.

### Impact of hit compounds on the conformational dynamics of KRAS^G12D^

The three hit compounds identified from the zinc database were further subjected to verification through molecular dynamics simulations. The four compounds were simulated for a period of 450 ns to reveal possible molecular events that underscore their binding to KRAS^G12D^. Unlike molecular docking that only proffer analysis on static complexes, molecular dynamics provides the opportunity to assess the events of the complexes over a period of time mimicking real-life situations. The complexes involving the compounds; ZINC78453217, ZINC70875226, and ZINC64890902 were therefore simulated. The compounds stability within the binding site and their permeation into the hydrophobic core of the protein together with their impact on the stability of the binding site as well as the global conformation dynamics of KRAS^G12D^ upon ligation were investigated.

### Hit compounds penetrates deeper into the hydrophobic core of KRAS^G12D^ with resultant stability

**Figure 8:**
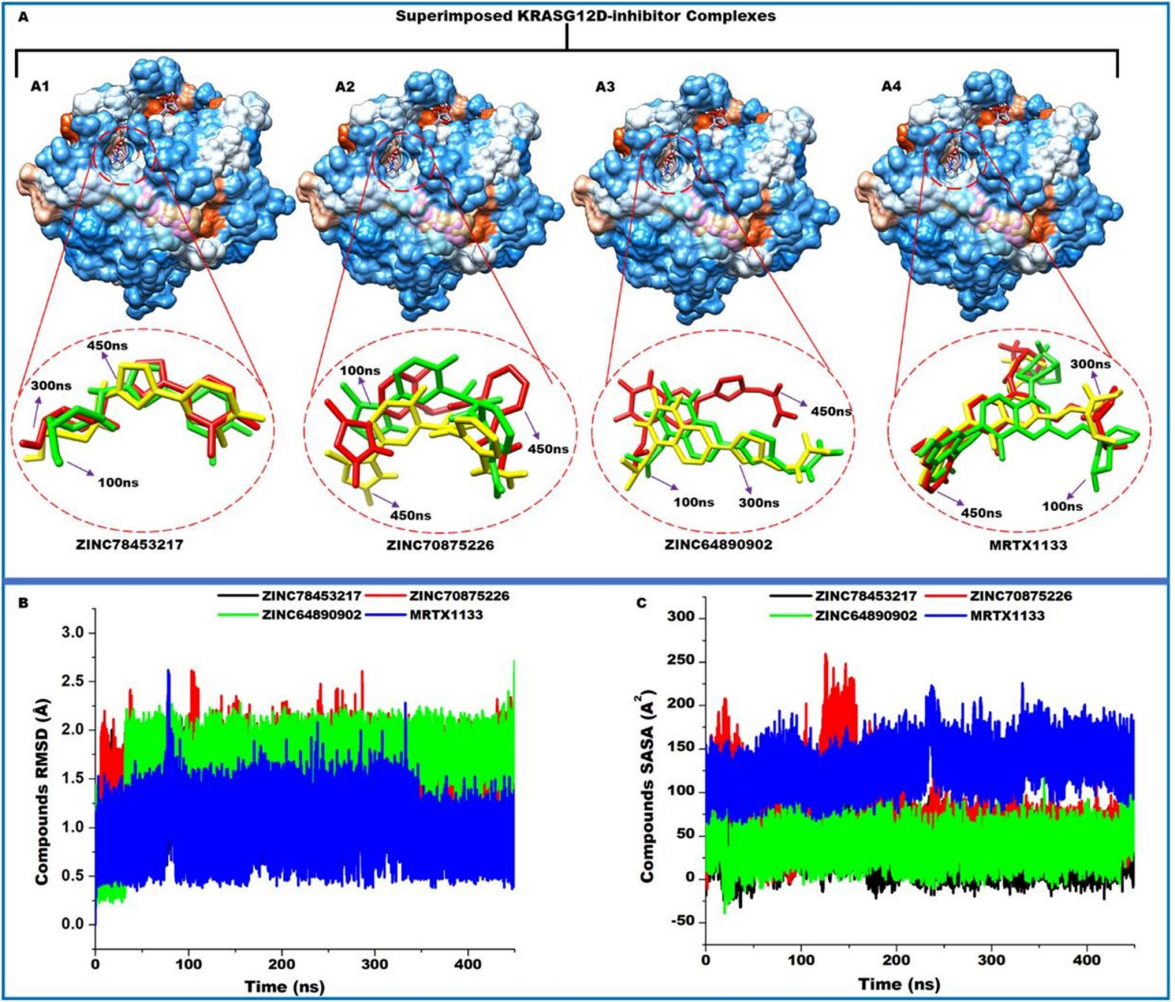
(**A**) Superimposition of KRAS^G12D^ complexed with ZINC78453217, ZINC70875226, ZINC64890902 and MRTX1133. Insert shows the binding orientation of each inhibitor at 100 ns, 300 ns, and 450 ns. (**B**) Comparative inhibitor stability plots using RMSD metrics for ZINC78453217, ZINC70875226, ZINC64890902 and MRTX1133. (**C**) Comparative inhibitor SASA plots of ZINC78453217, ZINC70875226, ZINC64890902 and MRTX1133.

The solvent accessibility surface area (SASA) of the compounds was evaluated to ascertain the surface area available for aqueous interaction during the simulation period. This information provides insights into the level of penetration of the compounds into the hydrophobic core of the protein^66^. Deeper penetration will therefore decrease the surface area availability of the compounds and vice versa. The compounds presented average SASA values of 34.89 ± 15.37A^2^, 75.48 ± 36.56A^2^, and 128.08 ± 39.10A^2^ for ZINC78453217, ZINC70875226, and ZINC64890902 respectively. These results depict ZINC78453217 penetrated deeper into the hydrophobic core compared to the other compounds since it presented the least available solvent accessible surface area. Binding and permeation of a compound to a protein is conditioned by intermolecular interactions which consequently influences the stability of the compound within the binding pocket. As such, the stability of the compounds was estimated. The root mean-square deviation matric was utilized to compute the atomic deviations of the compounds during the 450 ns period of simulation. This matric is informative on the extent to which atomic positions vary over time which is indicative of the stability of the compounds. The compounds exhibited average RMSD values of 1.25 ± 0.17 Å, 1.55 ± 0.20 Å, and 1.84 ± 0.28 Å for ZINC78453217, ZINC70875226, and ZINC64890902 respectively while MRTX1133 presented 0.97 ± 0.12 Å. These results indicate general stability of the compounds within the binding site. As observed from the values and Fig. 8, ZINC78453217 which exhibited the least SASA, is the most stable among the hit compounds suggesting it could possess higher therapeutic potentials. Afterwards we sought to determine the impact of these compounds on the binding pocket.

**Figure 9:**
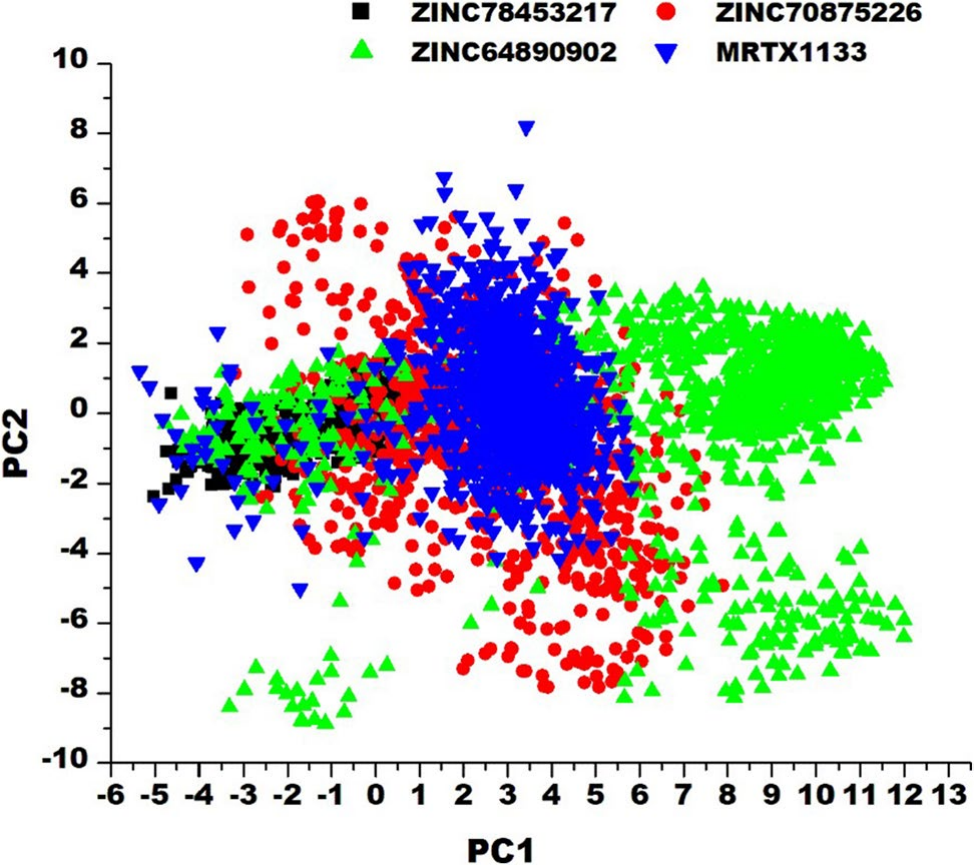
Principal component analysis of the motions of the selected compounds at the allosteric binding site of KRAS^G12D^.

The differential positioning and stability of the compounds relative to MRTX1133 was further ascertained by conducting the PCA of the compounds. We clustered the compounds movements in the direction of two principal components: PC1 and PC2. From this computation presented in Fig. 9, it is observed that the motions of ZINC64890902 over the simulation period is more dispersed than the other compounds including MRTX1133. ZINC78453217 was observed to display more compact motion during the simulation period while ZINC70875226 showed moderate dispersed motion relative MRTX1133. The compact motion exhibited by ZINC78453217 among the selected compounds corroborates with the RMSD and SASA findings wherein it showed the most stable with the least exposure to solvent environment among the compounds.

### Hit compounds disrupt binding site conformations

**Figure 10:**
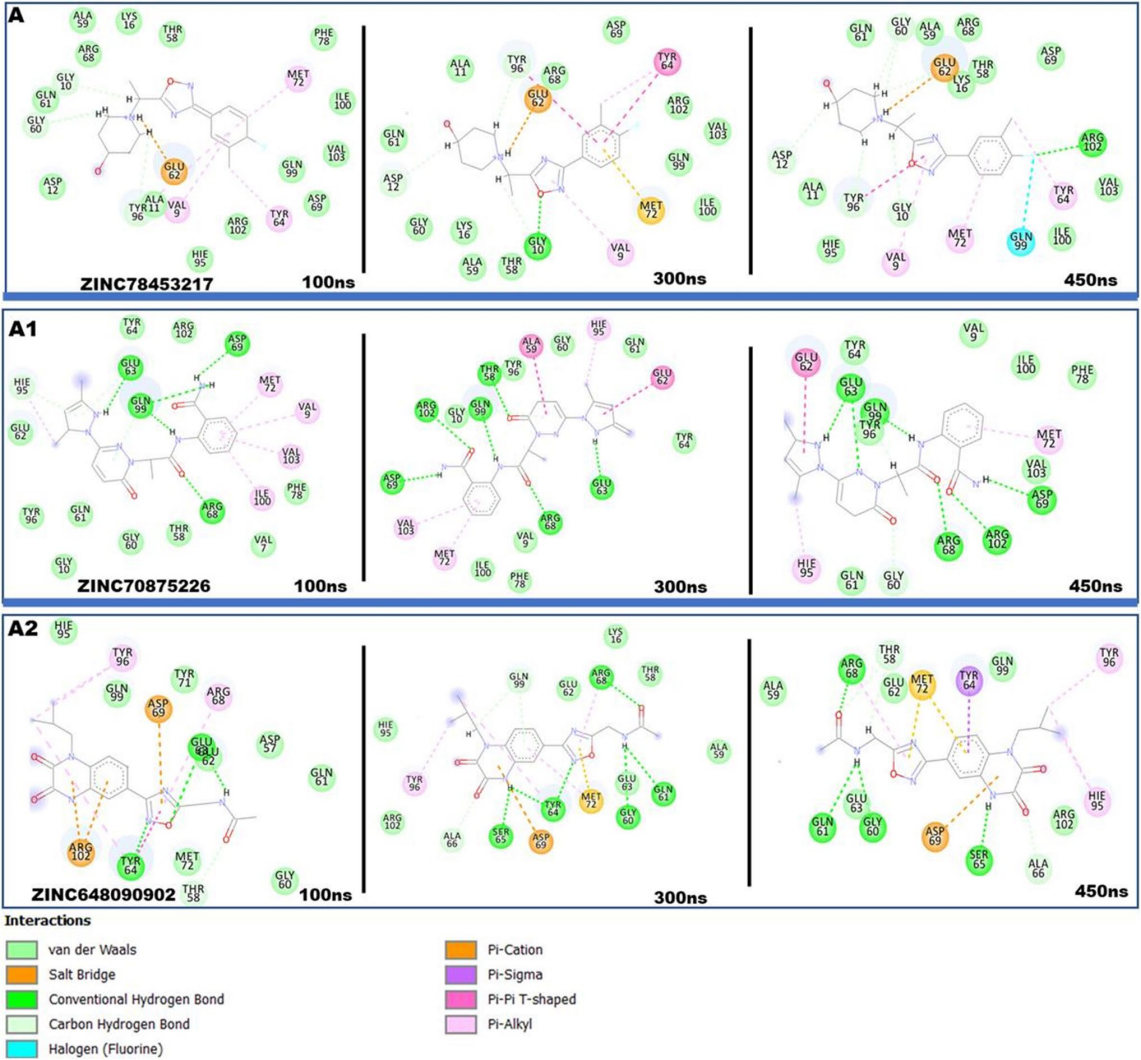
2D interactions of hit compounds with KRAS^G12D^ binding site residues. Snapshots depicts interactions at 100 ns, 200 ns, 300 ns and 450 ns. (**A**) Shows ZINC78453217 interactions with the binding site residues. (**A1**) Shows ZINC70875226 interaction with the binding site residues. (**A2**) Shows ZINC64890902 interactions with the binding site interactions.

**Figure 11:**
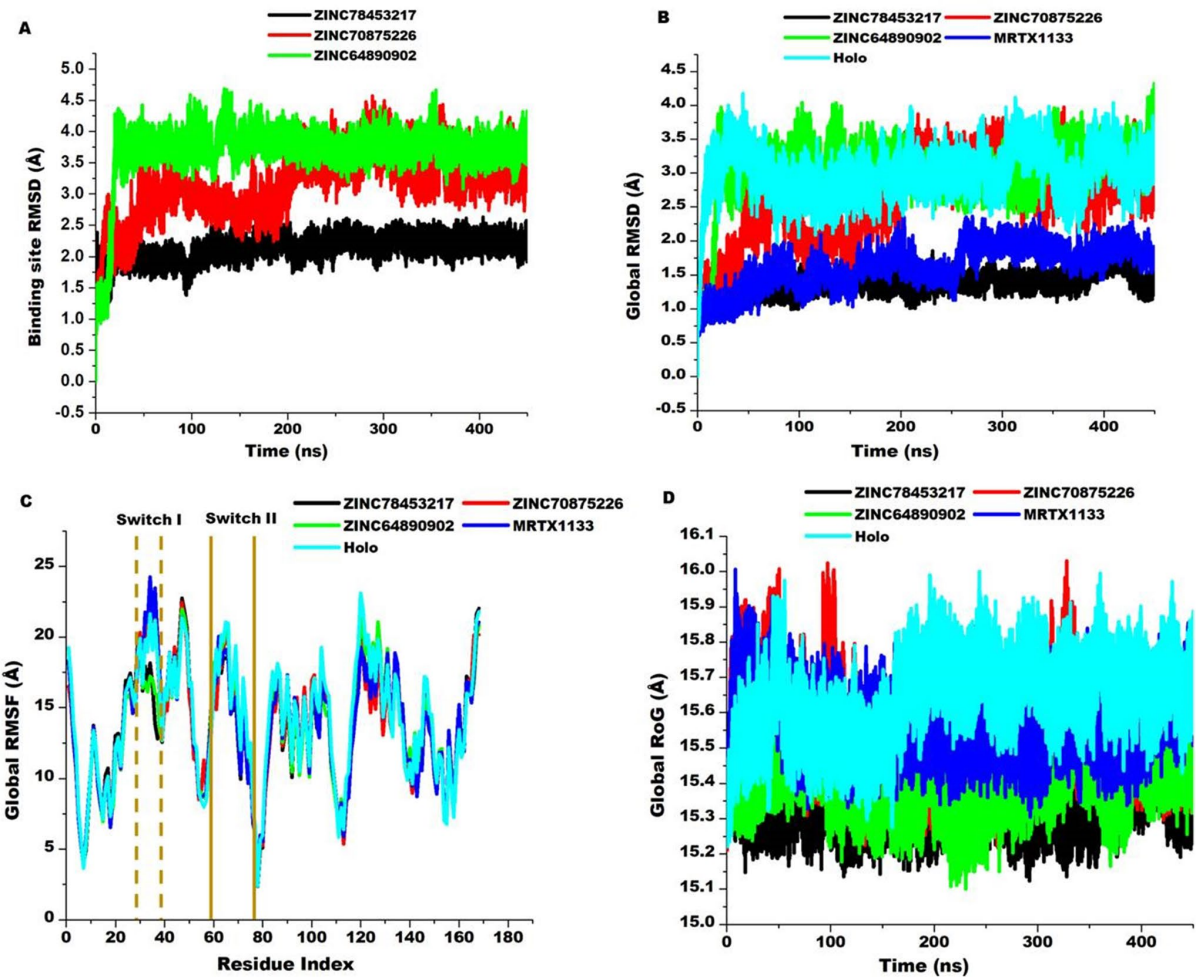
Comparative RMSD, RMSF and RoG plots of the hit compounds, MRTX1133 and unbound (holo) KRAS^G12D^ protein. (**A**) Shows the binding site RMSD elicited by the binding of the hit compounds. (**B**) Depicts the global stability of KRAS^G12D^ upon compounds binding. (**C**) Shows the residual fluctuations exhibited by the global protein residues during the simulation period. Highlights (deep yellow) show the switches regions. (**D**) Shows the RoG exhibited by the global protein. The bound conformations are contrasted to the unbound (Holo).

The inhibitory or activation ability of bioactive compounds is often underscored by conformational changes they induce on the target protein. Conformational changes beyond or below a certain threshold result in the disruption of the basal functionalities of the protein^67^. Thus, this phenomenon has been exploited by drug developers to contain certain abnormal protein activities. The effect of the compounds on the binding site of KRAS^G12D^ was therefore evaluated. Effects of compounds on the binding site and on the protein as a whole is often underscored by interactions that characterize the compound’s binding. We, therefore, visualized snapshots from the simulation and the results presented in Fig. 10. This revealed the compounds engaged in varying interaction types during the course of simulation. The RMSD of the C–α atoms of the binding site residues were determined. As Fig. 11 discloses, the compounds exhibited varying effects on the binding site. They presented average values of 2.12 ± 0.18 Å, 3.16 ± 0.48 Å, and 3.69 ± 0.46 Å, for ZINC78453217, ZINC70875226, and ZINC64890902, respectively. ZINC78453217 induced a stable binding site comparatively below MRTX1133 and the holo (Fig. 2) whiles the other compounds destabilized the binding site beyond the holo. These observations suggest the compounds elicit bioactivity on the KRAS^G12D^ binding pocket and therefore suggests their potentials as therapeutic agents.

### Hit compounds induces global stability with reduced flexibility of KRAS^G12D^

Proteins are sensitive to external stimuli and thus in most instances react through global conformational changes. These changes as earlier stated could influence the normal functions of the proteins. We, therefore, sought to determine the impact of the hit compounds on the entire KRAS^G12D^ through estimating the stability, flexibility and compactness. These was achieved through estimating the RMSD, the root mean-square fluctuation (RMSF) and the radius of gyration (RoG) of the C–α atoms of the protein over the 450 ns simulation period. While, the RMSD is informative of the general stability and system convergence over the period of simulation and therefore expresses the confidence in the ensuing deductions, the RMSF discloses the flexibility of the protein while RoG expresses the compactness of the protein. Higher RMSF values indicate more flexibility and vice versa while high RoG values indicate less compactness. As presented in Fig. 9, the systems presented average RMSD values of 1.38 ± 0.14 Å, 2.65 ± 0.56 Å, 3.00 ± 0.46 Å, 1.64 ± 0.13 Å and 3.46 ± 0.49 Å for the ZINC78453217, ZINC70875226, ZINC64890902, MRTX1133 and the holo respectively. The highest stability of the protein was observed with when ZINC78453217 was bound. This compound stabilized the protein below MRTX1133. Generally, the compounds induced elevated stability of the protein relative to the unbound. Probing the flexibility of KRAS^G12D^ showed a general reduction in the flexibility of the residues during the simulation period. Average RMSF values of 13.52 ± 4.05 Å, 13.65 ± 4.16 Å, 13.93 ± 4.33 Å, 13.73 ± 4.11 Å and 14.11 ± 4.36 Å for ZINC78453217, ZINC70875226, ZINC64890902, MRTX1133 and the holo protein, respectively. Zoning into the fluctuations that characterized the switches upon complexing with the compounds revealed ZINC78453217 and ZINC64890902 induced a reduction switch I residues fluctuations relative to the unbound. The other compounds together with MRTX1133 increased switch I fluctuation beyond the unbound. All the compounds however, reduced the fluctuations of Switch II residues below the unbound. The RoG also presented a similar trend of global protein behavior with the RMSF. A general reduction in the RoG values relative to the unbound was observed as presented in Fig. 11. This reduction indicates increased compactness and rigidity of KRAS^G12D^ upon the compounds binding corroborating the reduced fluctuations of the residues. Average RoG values of 15.34 ± 0.05 Å, 15.52 ± 0.11 Å, 15.40 ± 0.07 Å, 15.58 ± 0.08 Å, and 15.64 ± 0.10 Å for ZINC78453217, ZINC70875226, ZINC64890902, MRTX1133 and holo protein respectively.

### Binding energy landscape of hit compounds

The binding energy profile of the hit compounds were evaluated using the MM-PBSA method due to its popularity^68^. The binding energies are pivotal to ligand–protein complexing by disclosing the spontaneity, folding, permeation and kinetic reactions of the ligation process^50^. Higher binding affinity (most negative) indicates the strength of interactions involved in the ligation and thus reveals the potency of the compound. The hit compounds’ energy profiles were therefore determined and presented in Table 5. Average total binding free energies (ΔG) of − 38.69 ± 2.76 kcal/mol, − 41.80 ± 7.67 kcal/mol, and − 35.24 ± 3.99 kcal/mol were estimated for ZINC78453217, ZINC70875226 and ZINC64890902 respectively.

**Table 5:** Binding energy profile of hit compounds.

System	Energy components (kcal/mol)				
	$\Delta {\text{E}}_{{{\text{vdw}}}}$	$\Delta {\text{E}}_{{{\text{ele}}}}$	$\Delta {\text{G}}_{{{\text{gas}}}}$	$\Delta {\text{G}}_{{{\text{sol}}}}$	$\Delta {\text{G}}_{{{\text{bind}}}}$
ZINC78453217	− 41.23 ± 2.79	− 296.46 ± 12.51	− 337.69 ± 11.97	301.93 ± 11.97	− 35.76 ± 3.14
ZINC70875226	− 47.00 ± 5.87	− 41.62 ± 9.77	− 88.62 ± 13.51	46.82 ± 7.17	− 41.80 ± 7.67
ZINC64890902	− 40.35 ± 2.76	− 262.97 ± 24.03	− 303.33 ± 23.81	268.09 ± 21.92	− 35.24 ± 3.99

ΔE_ele_ = electrostatic energy; ΔE_vdW_ = van der Waals energy; ΔG_bind_ = total binding free energy; ΔG_sol_ = solvation free energy ΔG = gas phase free energy.

These results suggest spontaneous compounds complexing with KRAS^G12D^ with ZINC70875226 showing the highest energy. The variations in the binding free energies presented by the compounds could be due to the type of interactions that accompany their binding. Though these compounds generally presented lower total free binding energies compared to MRTX1133, nonetheless their impact on the protein indicates they elicit bioactive potentials and could therefore be optimized to improve their activity. Contributing significantly to the total free binding energies are electrostatic, van der Waals and the gas phase energies.

**Figure 12:**
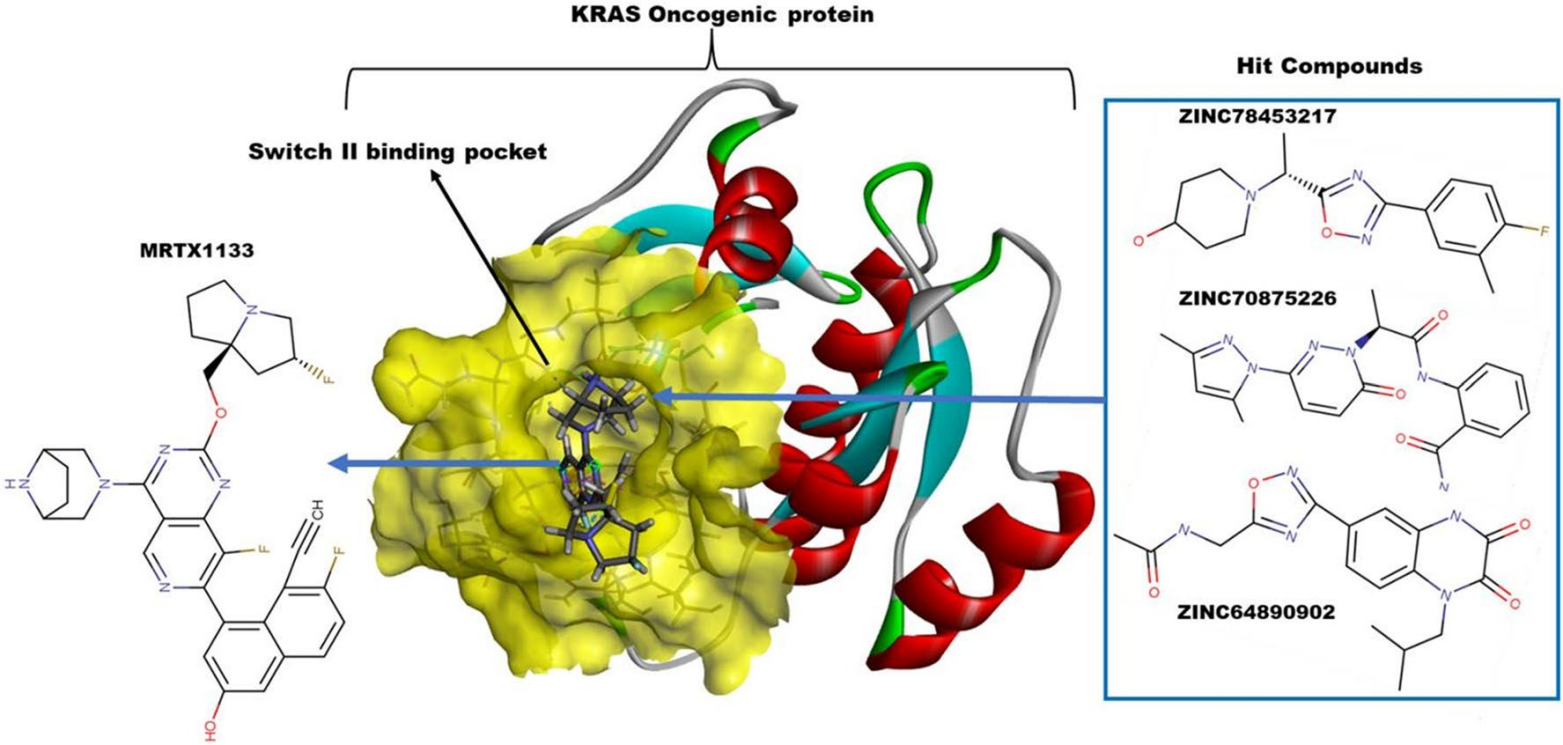
Most promising inhibitors of KRAS^G12D^.

To summarize the entire study, ZINC78453217, ZINC70875226 and ZINC64890902 are identified as the most promising inhibitors of KRAS^G12D^ (Fig. 12).

## Conclusion

Oncogenic KRAS has eluded drug developers for decades until covalent inhibition was strategically employed to target the mutated cysteine within the switch II binding pocket. Though this has achieved some success, cancers driven by other mutations has not been successful. The development of MRTX1133 to target KRAS^G12D^ has therefore been a sign of relief. In our quest to unravel the molecular events that highlight its potentials to inhibit KRAS^G12D^ as well as identify potential inhibitors based on its structural characteristics through PRED, we employed MD simulations and other computational techniques. The 450 ns simulation of MRTX1133 in complex with KRAS^G12D^ revealed MRTX1133 binding stabilized the binding site with a consequential reduction in the surface area of the site available for aqueous interaction. These changes resulted in a strong positive correlated movement of switch I and II which could disrupt KRAS^G12D^ interaction with effector and regulatory proteins. Again, the MRTX1133 was shown to exhibit strong binding affinity (− 73.16 ± 5.15 kcal/mol) with KRAS^G12D^ corroborating experimental findings. This energy was then decomposed into individual residue contributions revealing Asp69, His95, Met72, Tyr64, Gly60, Asp12 and Val9 contributed the highest towards the binding energy. A pharmacophore was subsequently developed based on the prominent moieties of MRTX1133 that the high-energy contributing residues interacted. The pharmacophore model was then employed to screen the ZINC database towards identifying potential KRAS^G12D^ binders. Through molecular docking, MD simulation and ADMET analysis, ZINC78453217, ZINC70875226 and ZINC64890902 were identified as the most promising binders of KRAS^G12D^. Further structural analysis through MD simulation showed the identified compounds particularly ZINC78453217 exhibited similar impact on KRAS^G12D^ through the stabilization of the global protein, reduction in flexibility and increase in compactness of the global protein. The successful application of multidisciplinary computational drug discovery approaches has allowed the identification of diverse potential therapeutic agents laying the ground work for experimental exploration of the suggested compounds. Further optimisations and validation of these compounds could aid in the discovery of effective KRAS^G12D^ inhibitors.

## Supplementary Information

Supplementary Information.

## Data Availability

All data generated or analyzed during this study are included in this published article [and its supplementary information files].
