# Supplementary material for: Characterization of the binding of MRTX1133 as an avenue for the discovery of potential KRASG12D inhibitors for cancer therapy
Source: Sci Rep. 2022 Oct 22;12:17796. doi: 10.1038/s41598-022-22668-1 (PMC9588042; doi:10.1038/s41598-022-22668-1)
Supplement: Supplementary file 1 — Supplementary Information. [file 41598_2022_22668_MOESM1_ESM.docx]

**Supplementary Information**

**Table S1.** Docking scores of selected compounds

| Entry Number | Canonical smile | Autodock Score (kcal/mol) | Glide HTVS Score (kcal/mol) |  |
| --- | --- | --- | --- | --- |
| ZINC40488765 | Cc1ccc(-c2noc([C@@H](C)N3CCC(O)CC3)n2)cc1F | -9.4 | -8.23 |  |
| ZINC92688822 | N#Cc1cc(Oc2ccc(C=O)cc2F)nc2ccc(Cl)cc12 | -9.3 | -7.92 |  |
| ZINC52961025 | Cc1ccc(-c2noc(CCCN3CC[C@@H](C(N)=O)C3)n2)cc1F | -9.5 | -8.39 |  |
| ZINC64890729 | CC(=O)NCc1nc(-c2ccc3c(c2)[nH]c(=O)c(=O)n3C(C)C)no1 | -9.1 | -8.94 |  |
| ZINC15731941 | Cc1cccc(-c2n[nH]c(SCc3cc(=O)n4oc(C)cc4n3)n2)c1 | -9.3 | -8.41 |  |
| ZINC19375312 | O=c1cc(CCCN2CCOCC2)nc(N[C@@H]2CCCc3ccccc32)[nH]1 | -9.7 | -8.32 |  |
| ZINC15731841 | CCc1nn2c(=O)cc(CSc3nc(-c4cccc(C)c4)n[nH]3)nc2s1 | -9.2 | -8.66 |  |
| ZINC09134721 | Cc1c(C)n(CCCO)c2ncn3nc(-c4ccc5ccccc5c4O)nc3c12 | -9.8 | -8.61 |  |
| ZINC19790710 | CN1CCC[C@H]1CCn1ccc(-c2cccc(-c3cnccn3)c2)n1 | -8.5 | -8.51 |  |
| ZINC04967953 | NC(=O)C1CCN(C(=O)CSc2nccc(-c3ccc(F)cc3)n2)CC1 | -8.5 | -8.40 |  |
| ZINC64890809 | CCCn1c(=O)c(=O)[nH]c2cc(-c3noc(CNC(C)=O)n3)ccc21 | -9.3 | -8.37 |  |
| ZINC31433679 | Cc1ccc2c(C)cc(SCC(=O)N3CCO[C@H](C)C3)nc2c1C | -8.7 | -8.19 |  |
| ZINC19794885 | CN1CCC[C@H]1CCn1ccc(-c2cccc(-c3cccnc3)c2)n1 | -8.6 | -8.15 |  |
| ZINC84280473 | Cc1nc(C(=O)N(C)[C@H]2CCS(=O)(=O)C2)nn1-c1ccc(F)cc1 | -8.8 | -8.12 |  |
| ZINC63646840 | Cc1ccc(-c2nnc(SCc3cc(=O)n4c(n3)S[C@@H](C3CC3)N4)[nH]2)cc1 | -9.2 | -8.08 |  |
| ZINC64950041 | Cc1ccc(-n2nc(-c3nc(-c4cccnc4)no3)c3ccccc3c2=O)cc1C | -9.3 | -7.97 |  |

**Table S2.** Physicochemical properties of selected compounds

| **ZINC**  **Compound ID** | **Formula** | **MW (g/mol)** | **MLog*P*** | **LogS (Ali) (mol/L)** | **TPSA (A^2^)** | **Molar Refractivity** | **HBA** | **HBD** | **Rotatable bonds** | **GI Absoption** | **Lipinski Drug likeness** |
| --- | --- | --- | --- | --- | --- | --- | --- | --- | --- | --- | --- |
| ZINC19790710 | C20H23N5 | 333.43 | 3.49 | -4.13 | 44.7 | 103.49 | 6 | 0 | 4 | High | Yes; 0 violation |
| ZINC63646840 | C18H18N6OS2 | 398.50 | 3.53 | -6.28 | 97.6 | 110.13 | 7 | 2 | 3 | High | Yes; 0 violation |
| ZINC52961025 | C21H24N2O | 332.37 | 1.02 | -2.47 | 96.7 | 90.88 | 7 | 2 | 5 | High | Yes; 0 violation |
| ZINC92688822 | C17H8ClFN2O2 | 326.71 | 2.72 | -4.92 | 82.1 | 83.33 | 5 | 0 | 4 | High | Yes; 0 violation |
| ZINC09134721 | C22H21N5O2 | 387.44 | 3.87 | -5.79 | 84.2 | 113.21 | 5 | 2 | 5 | High | Yes; 0 violation |
| ZINC15731841 | C17H16N6OS2 | 384.47 | 3.29 | -6.05 | 97.1 | 103.46 | 7 | 1 | 4 | Low | Yes; 0 violation |
| ZINC15731941 | C17H15N5O2S | 353.39 | 2.94 | -5.64 | 96.0 | 95.24 | 6 | 1 | 3 | High | Yes; 0 violation |
| ZINC84280473 | C15H17FN4O3S | 352.38 | 0.84 | -2.73 | 93.1 | 85.45 | 9 | 0 | 2 | High | Yes; 0 violation |
| ZINC64890809 | C16H17N5O4 | 343.34 | -0.63 | -2.47 | 148.9 | 90.58 | 11 | 2 | 4 | High | Yes; 0 violation |
| ZINC40488765 | C16H20FN3O2 | 305.35 | 1.96 | -3.13 | 64.5 | 84.32 | 6 | 1 | 3 | High | Yes; 0 violation |
| ZINC19375312 | C21H28N4O2 | 368.47 | 2.45 | -3.10 | 75.6 | 110.71 | 7 | 2 | 6 | High | Yes; 0 violation |
| ZINC04967953 | C18H19FN4O2S | 374.43 | 1.63 | -3.22 | 98.1 | 100.60 | 7 | 2 | 5 | High | Yes; 0 violation |
| ZINC64950041 | C23H17N5O2 | 395.42 | 3.83 | -6.15 | 87.7 | 113.80 | 6 | 0 | 0 | High | Yes; 0 violation |
| ZINC64890729 | C16H17N5O4 | 343.34 | -0.59 | -2.59 | 146.7 | 90.58 | 11 | 2 | 3 | High | Yes; 0 violation |
| ZINC19794885 | C33H31F3NO2 | 600.63 | 4.72 | 5.43 | 86.64 | 171.82 | 10 | 2 | 5 | High | Yes; 0 violation |
| ZINC31433679 | C19H24N2O2S | 344.47 | 3.37 | -4.30 | 44.9 | 103.49 | 5 | 0 | 3 | High | Yes; 0 violation |
| MRTX1133 | C29H41F2N5O | 513.67 | 4.15 | -6.22 | 86.64 | 145.84 | 7 | 1 | 9 | High | Yes; 1 violation |
